# Safety and Efficacy of 5-Aminolevulinic Acid for High Grade Glioma in Usual Clinical Practice: A Prospective Cohort Study

**DOI:** 10.1371/journal.pone.0149244

**Published:** 2016-02-17

**Authors:** Pilar Teixidor, Miguel Ángel Arráez, Glòria Villalba, Roser Garcia, Manel Tardáguila, Juan José González, Jordi Rimbau, Xavier Vidal, Eva Montané

**Affiliations:** 1 Department of Neurosurgery, Hospital Universitari Germans Trias i Pujol, Badalona, Barcelona, Spain; 2 Department of Neurosurgery, Hospital Carlos Haya, Málaga, Spain; 3 Department of Neurosurgery, Hospital del Mar, Barcelona, Spain; 4 Department of Neurosurgery, Hospital Clínic I Provincial de Barcelona, Barcelona, Spain; 5 Fundació Institut Català de Farmacologia, Hospital Universitari Vall d’Hebron, Barcelona, Spain; 6 Department of Pharmacology, Therapeutics and Toxicology, Universitat Autònoma de Barcelona, Barcelona, Spain; 7 Department of Clinical Pharmacology, Hospital Universitari Germans Trias i Pujol, Badalona, Barcelona, Spain; Cedars-Sinai Medical Center, UNITED STATES

## Abstract

**Background:**

During the last decade, the use of 5-aminolevulinic acid (5-ALA) has been steadily increasing in neurosurgery. The study's main objectives were to prospectively evaluate the effectiveness and safety of 5-ALA when used in clinical practice setting on high-grade gliomas’ patients.

**Methods:**

National, multicenter and prospective observational study. Inclusion criteria: authorized conditions of use of 5-ALA. Exclusion criteria: contraindication to 5-ALA, inoperable or partial resected tumors, pregnancy and children. Epidemiological, clinical, laboratory, radiological, and safety data were collected. Effectiveness was assessed using complete resection of the tumor, and progression-free and overall survival probabilities.

**Results:**

Between May 2010 and September 2014, 85 patients treated with 5-ALA were included, and 77 were suitable for the effectiveness analysis. Complete resection was achieved in 41 patients (54%). Surgeons considered suboptimal the fluorescence of 5-ALA in 40% of the patients assessed. The median duration of follow-up was 12.3 months. The progression-free survival probability at 6 months was 58%. The median duration overall survival was 14.2 months. Progression tumor risk factors were grade of glioma, age and resection degree; and death risk factors were grade of glioma and gender. No severe adverse effects were reported. At one month after surgery, new or increased neurological morbidity was 6.5%. Hepatic enzymes were frequently increased within the first month after surgery; however, they subsequently normalized, and this was found to have no clinical significance.

**Conclusion:**

In clinical practice, the 5-ALA showed a good safety profile, but the benefits related to 5-ALA have not been yet clearly shown. The improved differentiation expected by fluorescence between normal and tumor cerebral tissue was suboptimal in a relevant number of patients; in addition, the expected higher degree of resection was lower than in clinical trials as well as incomplete resection was not identified as a prognostic factor risk for death. Because optimal fluorescence was correlated to higher complete resection rate, further research is needed to identify patients (or tumors) with more surgery benefits when using the 5-ALA.

## Introduction

Malignant cerebral gliomas are the most frequent type of primary cerebral tumor, as well as the most lethal.[1,2] They tend to be diagnosed in the sixth and seventh decades of life. Although there is a correlation between the extent of tumor resection and survival, data analysis is controversial.[3,4] Complete tumor resection increases the efficacy of adjuvant treatments, such as chemotherapy and radiotherapy.[5,6] Unfortunately, the infiltrating nature of malignant gliomas makes complete cytoreduction very difficult for the neurosurgeon, as the tumor margin is easily confused with normal healthy tissue.

During the last few years, new techniques have been developed to improve the degree of resection. These include: neuronavigation, intra-operative echography, intra-operative MRI, and the use of 5-aminolevulinic acid (5-ALA). The 5-ALA is a biochemical precursor from the heme group. It is administered orally leading to an accumulation of fluorescent protoporphyrin in the malignant glioma.[7] This fluorescence can be visualized during the operation using a modified microscope, and allows the neurosurgeon to differentiate normal cerebral tissue and tumor tissue.

The 5-ALA was approved by the European Medicines Agency (EMA) as an orphan drug in 2007. On the contrary, in the United States, it has not been yet approved by the Food and Drug Administration (FDA); presently, many studies are being carried out (data available in https://clinicaltrials.gov/). The pivotal clinical trial which aimed to evaluate the efficacy of 5-ALA in 322 patients, was promptly terminated due to the beneficial results in the interim analysis (with 270 patients included). Complete resection on MRI was achieved in 65% of the patients treated with 5-ALA, compared to 36% of patients receiving conventional surgery with white light.[8]

The 5-ALA effectiveness in usual clinical practice has been widely assessed in retrospective cohort studies but not in prospective studies.[9–11] We want to point out that in the available systematic reviews and/or metaanalysis studies related to intraoperative 5-ALA in high grade gliomas, the efficacy data assessed were obtained from clinical trials and observational studies together.[12,13] It is widely known that clinical trial conditions are strongly different from clinical practice setting, leading to relevant differences in the obtained results.[14] To our knowledge, this is the first prospective cohort study assessing the safety and the effectiveness of the 5-ALA used in multiple center clinical practice setting.

The main objectives of this study were to prospectively evaluate the effectiveness and safety of 5-ALA when used in clinical practice setting on high-grade gliomas’ patients.

## Materials and Methods

We followed the STROBE Statement to report the study sections and their content.[15] (S1 STROBE Checklist)

In order to increase the sample size, an extension of the study was carried out; this extension included the addition of a new participating center, the subjective assessment of the fluorescence intensity of 5-ALA by the neurosurgeons, and the extension of the duration of the study to two years. The methods and results shown are from the study and its extension altogether.

### Study design and patients

This is a multicenter prospective cohort study of patients with high grade glioma treated with 5-ALA before surgery. It was performed at four hospitals in Spain provided with a neurosurgery service: Hospital Germans Trias i Pujol (Barcelona), Hospital del Mar (Barcelona), Hospital Clínic (Barcelona), and Hospital Carlos Haya (Malaga). The ethics committee of the coordinating centre (Comitè Ètic d’Investigació Clínica de l’Hospital Germans Trias I Pujol) approved the study in April 2010 and its extension in May 2012. Other ethics committees of participating centres approved the study (such as Comité Ético de Investigación Clínica IMAS, Hospital del Mar). Eligible patients were those who met the current authorized use for 5-ALA. Inclusion criteria were over 18 years of age, presumed radiological diagnosis of grade III or IV glioma according to WHO classification, and forecast of complete or nearly complete surgical resection. Exclusion criteria were any contraindication to 5-ALA, pregnancy, non-surgical removal of the tumor, or forecast partial resection. All patients were informed about the study and signed the informed consent before participating in the study. The recruitment study period was from May 2010 to September 2014. The study ended in December 2014.

### Pharmacological treatment

The drug (Gliolan^®^ Medac, Wedel, Germany) was administered according to the product information.[16] The administered dose was 20 mg/kg bodyweight. This was dissolved in water (1,500 mg of 5-ALA into 50 ml of water) and delivered orally approximately 4 hours before general anesthesia. In order to avoid severe skin reactions related to 5-ALA, the skin of the patients was protected from the effects of UVA light and sunglasses were handed to the patients during 24 hours after surgery.

### Surgical procedure

Surgery was performed using standard microsurgical techniques. The microscope was switched from conventional white xenon illumination to violet-blue excitation light to allow visualization of tumor fluorescence during resection. Intra-operative neuronavigation was used in all cases; nevertheless intra-operative ultrasound was still used in some cases. Neurosurgeons assessed the visible fluorescence during tumor surgery resection.

### Endpoints

The main endpoints of the study were drug effectiveness and drug safety.

The effectiveness endpoint was measured through the proportion of patients with histologically confirmed malignant glioma and complete resection of the tumor. Patients without histologically confirmed high-grade glioma were excluded from the analysis. The volume of the residual tumor was measured using contrast MRI performed within 5 days of surgery. The volume was calculated using manual segmentation of the tumor outline across all sections of the MRI scan. In each center, a specialist neuroradiologist, used the same method performed this calculation. Complete resection was defined as no contrast enhancement or resection of more than 95% of the initial tumor volume, otherwise was defined as non-complete resection.

The safety endpoint was assessed globally with any reported adverse events, analytical disorders, and/or neurological deficits after surgery. Hematological, liver, and renal function were assessed with a laboratory blood test, extracting a sample before surgery and at 24 hours, 48 hours, 5 days, 1 and 3 months after surgery. Neurological evaluation was used to assess clinical status before surgery and 1, 3, and 12 months after surgery. New postoperative neurological deficits identified at 1-month post-surgery were regarded as temporary if they had completely recovered at 3 months post-surgery and as permanent if they persisted at 3 months post-surgery.

Other effectiveness endpoints were overall survival (OS) and progression-free survival (PFS) probabilities assessed at 6, 9, 12, and 24 months post-surgery. The Karnofsky Performance Scale (KPS) was assessed before surgery.

### Study size

The estimated size of the study was about 5 patients/year in each center; therefore approximately 100 patients were considered to be included in the study.

### Statistical analysis

Because of skewed distributions, median, interquartile range (IQR), and range were used to describe continuous clinical variables.

Inferential methods were used to estimate statistical significance from variable associations, especially from the primary endpoint against the secondary endpoints. For contingency tables and for categorical variables, exact Pearson’s χ^2^ test was used for comparison, and for continuous variables, Wilcoxon rank sum test.

Survival analysis was performed using the Kaplan-Meier method, estimating the time from surgery to the point of disease progression (duration of PFS) and death (duration of OS). To compare survival between groups defined by the primary endpoint (complete resection or incomplete resection), the log-rank test was used when the assumption of proportional risk was met and the Wilcoxon test was used when this assumption was breached. The Cox proportional risk method was used to estimate the hazard ratio (HR) between the variable categories, after adjusting for confounding factors. This was the method followed after graphically verifying the assumption of proportional risks related to the test validity. Statistical significance was set at p<0.05. Statistical analyses were performed with SAS^®^ 9.3 (SAS Institute Inc., Cary, NC, USA).

## Results

Between May 2010 and September 2014, 85 patients treated with 5-ALA were included in the study. However, eight patients were subsequently excluded from the effectiveness analysis because the histology did not confirm a high-grade glioma (oligodendroglioma grade II n = 3, astrocitoma grade II n = 2, metastasis n = 2, and meduloblastoma n = 1).

### Clinical descriptive analysis

Of the remaining 77 patients included in the analysis, 43 were men (55.8%) and the mean (SD) age was 57.7 years (13.8); IQR 22, range 23–79 years. The baseline clinical characteristics are shown in Table 1. Co-morbidity, such as diabetes, hypertension, cardiomyopathy, or other cancers, was present in 51 patients (66.2%).

**Table 1:** Base-line characteristics of the included patients.

	Included patients(n = 77)
**Men**	43 (55.8%)
**Age (years)** Median (range)	57.7 (23–79)
>55	51 (66.2%)
**Co-morbidity**	51 (66.2%)
**Karnofsky performance scale**	
<70	4 (5.1%)
70–80	11 (14.1%)
>80	63 (80.8%)
**Tumor location**	
Non-eloquent	41 (52.6%)
Eloquent	36 (47.4%)
**Size tumor (cm)**	
≤5	50 (65%)
>5	27 (35%)
**Shinoda score**	
≤2 points (stage I)	46 (60.5%)
3 points (stage II)	19 (25%)
≥ 4 points (stage III)	11 (14.5%)
**Histology**	
Grade III	11 (14.3%)
-Anaplastic astrocytoma	6
-Oligodendroglioma	3
-Oligoastrocytoma	2
Grade IV	66 (85.7%)
-Glioblastoma multiforme	65
-Gliosarcoma	1

Preoperative MRI indicated that most tumors were unifocal (75 patients, 97.4%), had small size (≤5 cm) (50 patients, 65%), were located in a non-eloquent brain area (41 patients, 53.2%), and were classified as type I (46 patients, 60.5%) or type II (19 patients, 24.7%) according to Shinoda classification. The histology analysis showed 11 patients (14.3%) with grade III glioma and 66 (85.7%) with grade IV glioma (Table 1).

The main neurological signs and symptoms before surgery were focal disorders (hemiparesis, aphasia or visual alterations), that were present in 39 patients (50.6%), and convulsions in 22 (28.6%). Cognitive impairment at diagnosis was present in 39 patients (50.6%). High intracranial pressure was detected in 29 patients (37.7%).

The preoperative KPS score ranged from 30% to 100%, and was <80% in 15 patients (19.5%).

### Tumor resection

Complete resection was achieved in 41 patients (53.9%). No significant statistical associations were found for resection degree (complete vs incomplete) with age, gender, hospital centre, co-morbidities presence, preoperative KPS score, tumor size, tumor location in an eloquent brain area, and tumor histological grade.

The MRI was performed within 3 days of surgery in 59 patients (77.6%); on the fourth day in 11 patients (14.5%), and between the fifth and the seventh day in 6 patients (7.9%).

Assessment of 5-ALA fluorescence: based on subjective impression of the surgeon, the visual fluorescence of the 5-ALA was suboptimal in 40.6% of patients (13/32); on those 13 patients, the fluoresecence was of low intensity in 11, and none detected in two patients. Complete resection rate was higher in patients with optimal fluorescence than in those with suboptimal (73.7% vs 22.2%; p = 0.0293).

Duration of surgery resection: the mean (SD) duration of the surgical procedure was 4.5 hours (57 minutes); ranging from 2.75–8 hours.

### Safety

All treated patients were assessed on the safety analysis regarding adverse events. No cardiovascular, gastrointestinal, or skin effects were reported. No severe or life-threatening adverse effects were reported. For the analytical disorders and neurological deficits after surgery, all included patients were assessed.

Regarding analytical disorders, changes in the blood count and hepatobiliary enzymes were reported. No deterioration of renal function neither coagulation disorders were reported.

- Hematological disorders were frequent, but were mild and promptly recovered spontaneously. These included leukocytosis, anemia and thrombocytopenia.
- A mild and asymptomatic increase of the hepatobiliary enzymes was described. Patients with isolated and no clinically relevant elevations of hepatic enzymes before surgery were at risk to further increases. Alanine aminotransferase (ALT) and gamma glutamyl transferasa (GGT) were the most frequently increased. Transaminases (AST and ALT) and bilirubin increased at 24 hours post-surgery and GGT at day 5, and all of them decreased progressively within the following month. Alkaline phosphatase was not altered. At 24 hours post-surgery, the amylase enzymes values were abnormal in 34% of patients (maximum: 1,232 U/L), but progressively normalized within the following month. (Table 2).

**Table 2:** Summary of hepatobiliary disorders.

	Maximum % of patients with increased value	Time from drug administration	Peak or Maximum value (U/L)
**AST**	19.2	24 hours	207
**ALT**	36.5	24 hours	472
**GGT**	37.7	Day 5	899
**Bilirubin**	5.4	24 hours	71 (μm/L)
**Amylase**	34	24 hours	1.232

AST and ALT: transaminase; GGT: gamma glutamyl transferasa

Referring to neurological effects, 14 patients (18.2%) presented with some degree of a new onset of a neurological deficit or worsened the previous detected on the preoperative visit. The most frequent deficit was the language impairments. Neurological deficit persisted in 5 patients (35.7%) during the follow-up period, mainly on those with motor and/or a sensitive deficit. On the contrary, all patients with seizures or status epileptic after surgery, recovered with anticonvulsant treatment. One month after surgery, the rate of neurological morbidity was 6.5%. All these patients except five (64.3%) had tumor localized in an eloquent area).[17]

### Survival analysis

The mean (SD) duration of follow-up was 12.3 months (7.3) (95% CI 5.7–17.7; IQR 12.01; range 4 days–33.6 months).

**Fig 1:**
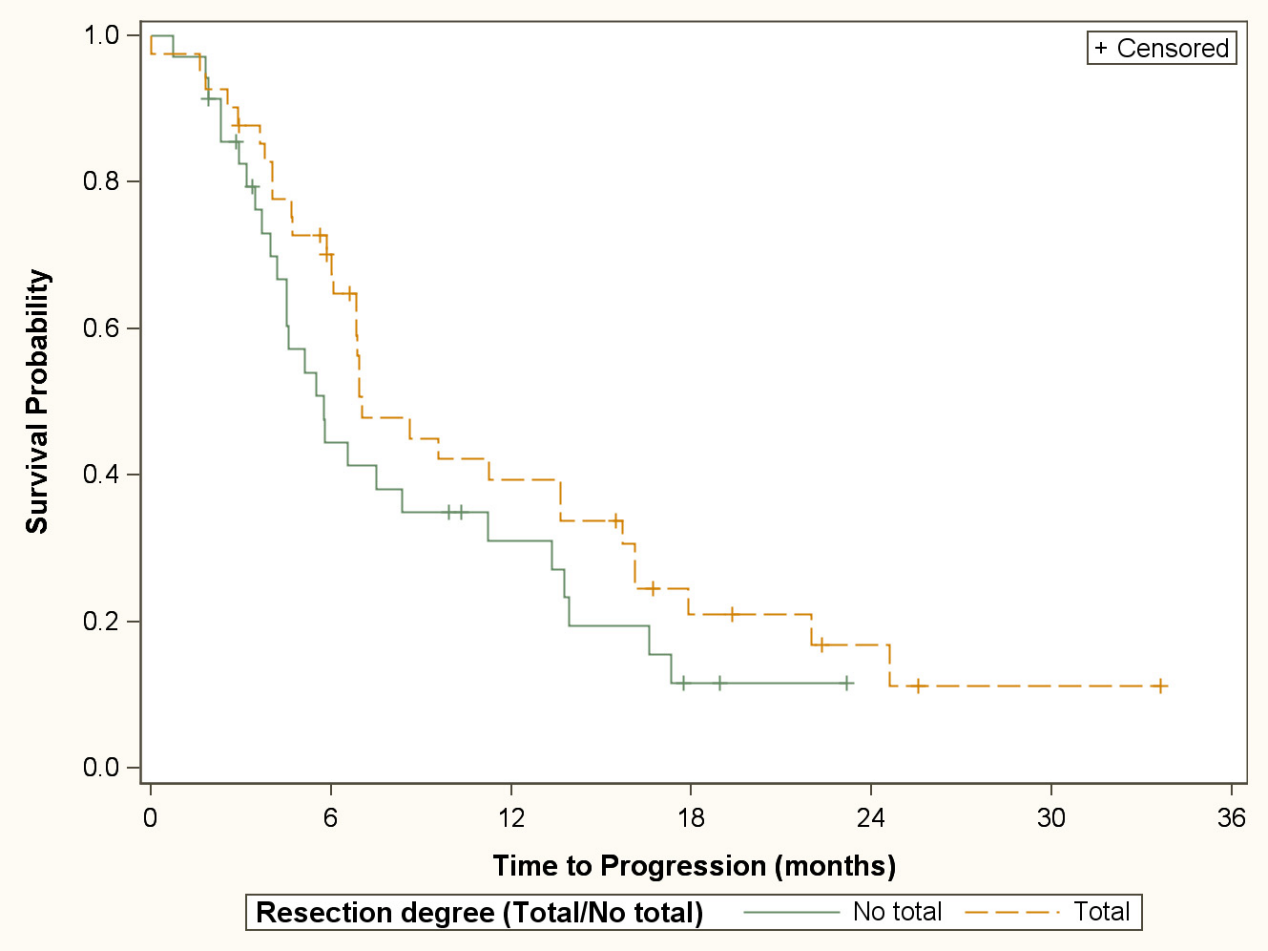
Cumulative survival probability for tumor progression according to resection degree.

The median duration of PFS was 6.9 months (95% CI 5.7–9.6). PFS probability at 6, 9, 12 and 24 months was 58% (41 patients), 40% (27 patients), 35% (22 patients), and 14% (5 patients) respectively. The degree of resection (complete or incomplete) was not statistically significant associated with the PFS (median PFS: 7.0 months [95% CI 6.1–13.6] *vs*. 5.7 months [95% CI 4.2–11.2]; p = 0.17) (Fig 1). Cox proportional hazards model showed that patients with IV grade glioma, older than 55 years, and incomplete resection degree, had an increased risk of progression in the follow-up period (Table 3).

**Table 3:** Results in a multivariate Cox proportional hazards analysis on PFS.

Variable		Hazard Ratio (95% Confidence Limits)	P Value
**Age > 55+**	**55**	2.05 (1.12–3.75)	0.020
**Tumor degree**	**Grade IV**	2.92 (1.15–7.41)	0.024
**Resection degree**	**Incomplete**	1.74 (1.01–2.98)	0.045

**Fig 2:**
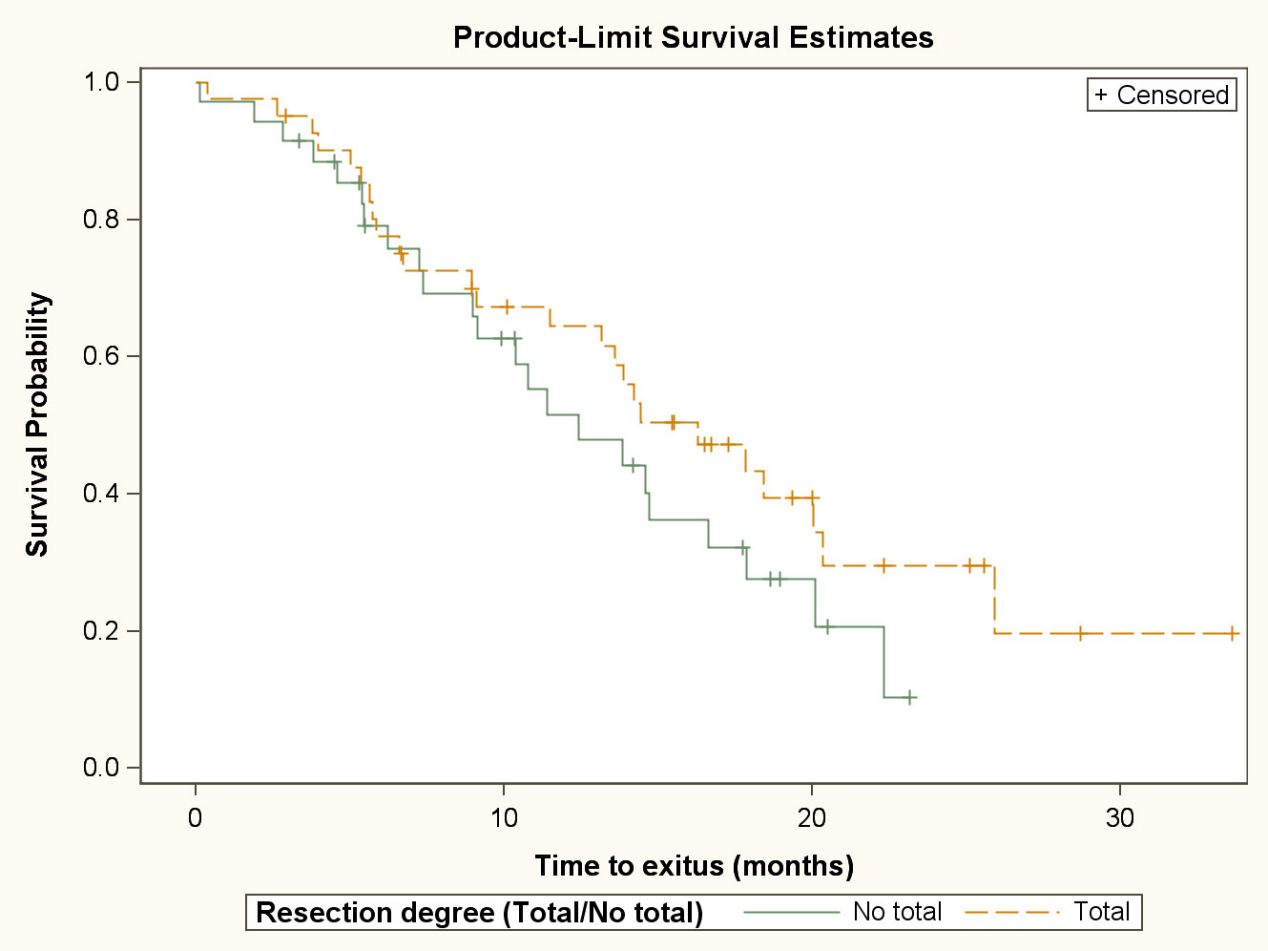
Cumulative survival probability for death according to resection degree.

The median duration of OS was 14.2 months (95% CI 11.4–17.8). OS probability at 6, 9, 12, and 24 months was 78.4% (56 patients), 68.5% (47 patients), and 59.3% (38 patients), and 22% (7 patients), respectively. The degree of resection (complete or incomplete) was not statistically significant associated with the OS (median OS: 16.3 months [95% CI 11.5–20.3] *vs*. 12.4 months [95% CI 9.0–16.6], p = 0.24) (Fig 2). Cox proportional hazards model showed that IV grade gliomas and men had an increased risk of death in the follow-up period. (Table 4)

**Table 4:** Results in a multivariate Cox proportional hazards analysis on OS.

Variable		Hazard Ratio (95% Confidence Limits)	P Value
**Gender**	**Male**	2.03 (1.08–3.77)	0.027
**Tumor degree**	**Grade IV**	3.51 (1.08–11.46)	0.038

### Surgical complications

Five patients (6.5%) undergo a surgical complication, probably not related to the 5-ALA use. Four of them (80%) recovered and one with cerebral edema and coma was resubmitted to surgery but death within the following three months.

### Post-hoc analysis of complementary treatment

Post-hoc exploratory analysis comparing patients treated according to the Stupp protocol,[18] with other therapies than surgery such as radiotherapy or chemotherapy (temozolomide) were done. There were no statistically significant differences in the PFS or in the OS when comparing patients treated with all therapies (surgery + radiotherapy + chemotherapy) to patients treated with surgery and radiotherapy or chemotherapy (p = 0.447 and p = 0.632 respectively).

## Discussion

Since the EMA's approval, the use of 5-ALA has been increasing in neurosurgery, although its use requires the neurosurgical microscope to be adapted and the neurosurgeon to be the accredited.[16]

The most important findings in this study were 1) 5-ALA fluorescence was suboptimal in 40% of the assessed patients; 2) complete resection was achieved in 54% of patients; 3) PFS probability at 6 months was 58% and the median duration OS was 14.2 months; 4) the high grade of the gliomas (grade IV) is a predictor factor risk of progression and death; and 5) 5-ALA has good safety profile.

The pivotal study by Stummer et al. provided the highest level of evidence assessing the efficacy of 5-ALA,[8] therefore, its main efficacy results are considered as a reference when comparing to the present study.

1 The neurosurgeons considered the fluorescence visually suboptimal in 40% of the patients assessed, similar to other studies, but this was lower when using the quantitative fluorescence measurements.[19] The fluorescence assessment was added in the extension study and only a third of the included patients were evaluated. However, we would like to point out that complete resection rate was higher in patients with optimal fluorescence than those with suboptimal fluorescence. On one hand, this assessment is subjective and shows an impression of surgeon. On the other hand, the intensity of the fluorescence could be variable attending to the grade or molecular characteristics of the tumor; as well as pharmacokinetics features of the patients. At present, we are not able to identify before surgery patients whose fluorescence will be optimal to increase drug benefits.
2 The proportion of patients with complete resection in the present study was lower than in the pivotal study (54% *vs*. 63%).[8] This is probably due to several reasons. Firstly, the expertise of the surgeons may have influenced this outcome. The intra-operative use of fluorescence needs to be learned by the neurosurgeon. There are different shades of fluorescence that are subjectively evaluated; therefore, the proficiency of the surgeon increases with experience. In our study, the procedures were performed by different neurosurgeons, with different levels of experience, and located in different hospitals. Secondly, in the presented study there may have been an overuse of 5-ALA by the neurosurgeons in the context of 5-ALA is only approved for patients with high-grade glioma with the possibility of nearly complete or complete resection. However, there is evidence that the neurosurgeon’s perception of the tumor does not always represent reality. Orringer et al. suggested that only a third of glioblastoma patients for whom the neurosurgeon predicted complete resection achieved this radiologically.[20] Thirdly, in one out of five patients the MRI was performed after the third day of surgery; mainly due to patients’ clinical situation, or the inability to perform MRI in the optimal period. Afterwards, that is, from the fourth day after surgery, non-neoplastic contrast enhancement due to surgical manipulation becomes radiologically apparent.[21] Finally, we did not record the use of complementary intra-operative techniques, such as echography and neuronavigation. Intra-operative techniques that improve the degree of resection have been developed in the last two decades. However, the availability of each of these techniques differs across neurosurgery departments and is changing over time. Therefore, the efficacy of each technique is difficult to compare between hospitals. This is one of the presented study’s limitations, meaning that we are not able to compare results according to the use of complementary intra-operative techniques. However, our results for complete resection is similar to several other reports of 5-ALA efficacy that have not reported the use of other intra-operative techniques in high-risk glioma resection.[8, 19, 22]
3 In contrast to the lower proportion of patients with complete resection, the PFS probability at 6 months was higher in this study than in the pivotal study (58% *vs*. 41%).[8] This result could be explained by the highest proportion of patients with gradeIII glioma in this study (13% *vs*. 2%),[8]) which have a lower recurrence rate than grade IV gliomas.[23] Nevertheless, the median duration of OS was nearly 14 months in both studies.[8] This could be explained because grade III and IV gliomas are both high-grade malignant.[23]
4 The grade of the gliomas (grade IV) as a predictor factor risk of progression and death is an expected find related to the malignancy of the tumor. Moreover, the fact that in the study most of the >55 years old patients (92%) and males (93%) had a glioma grade IV, could explain this result.

Differences in efficacy results (size effect) between clinical trials and observational studies have already been described; however it can be reduced when methodological high-quality studies are designed.[14]

5 Regarding the safety results, no cases of gastrointestinal, photosensitivity or hypotension occurred in our study, contrary to described in the Gliolan^®^ product information (in 0.1–1% of treated patients). In other studies, severe hypotension was present in almost 11% of patients, mainly in whom suffer from low blood pressure already.[16,24] Photosensitivity side effect was probably minimized by protecting the skin and eyes of patients from direct light during 24 hours after 5-ALA was administered, similarly to described in other studies.[9]

Other side effects described were liver function and hematological disorders (10% of treated patients).[16] More than 30% of patients had a hepatobiliary disorder, comparing to 2% of another study;[24] this discrepancy may be due to differences in the definition of liver disturbance. As described in the product information, increase in the amylase range happened frequently.[16] Interestingly, we observed that patients with elevated hepatic enzyme levels before surgery needed closer monitoring post-surgery (weekly monitoring until hepatic enzyme recovery to normal range). In addition, note that avoiding hepatotoxic drugs in all treated patients to prevent this liver side effect is important. With this knowledge, these findings allowed us to establish a monitoring protocol for patients with previous liver or hematological disorders. In the same way as Stummer et al. and Wong et al., we also consider that blood cell count disorders could be more related to surgery rather than 5-ALA administration,[8,24] and the corticosteroids treatment could produce leukocytosis too.

Some of the observed neurological effects in the presented study could also be explained by the mere occurrence of surgery, rather than the use of 5-ALA. The incidence of neurological complications (6.5%) is similar to that reported in other prospective studies.[25] As expected, the majority of those patients had already risk factor to produce neurological deficit, some of them related to the tumor (such as size or localization), but also related to the surgery and/or to the surgeon.

### Limitations

By the nature of the design, the main limitation of this study is the absence of a control group, meaning that we cannot compare effectiveness and safety between patients who did and did not receive 5-ALA. Furthermore, the use of other intra-operative techniques was not recorded. The sample size of the study is also a limitation of the conclusions drawn.

On the other hand, the strength of this study is that it is the largest multicenter prospective study assessing the effectiveness and the safety of 5-ALA in patients with high grade glioma in a real clinical practice setting. Prospective studies are designed with specific data collection methods, and therefore may be more complete than retrospective studies. One disadvantage of a prospective cohort study is the long follow-up period required to wait for events or diseases to occur.[26] However, the latency period of gliomas is short and the risk of loss to follow-up is very small in this population. Descriptive cohort studies often follow small group of patients. Published studies with similar or bigger sample size to our study are retrospective and/or single centre.[9–11]

## Conclusions

In clinical practice, the 5-ALA showed a good safety profile, but the benefits related to 5-ALA have not been yet clearly shown. The improved differentiation expected by fluorescence between normal and tumor cerebral tissue was suboptimal in a relevant number of patients. In addition, the expected higher degree of resection was lower than in clinical trials, as well as incomplete resection was not identified as a prognostic factor risk for death; by contrary to glioma grade. The present conclusions require confirmation in large prospective and multicenter studies. Combining different surgery tools is mandatory to better identify tumor tissue and achieve a complete and safer resection. The results of this study suggest that the 5-ALA is not an essential tool for high grade glioma resection, but could add some value to other tools in guiding resection for some patients. Because optimal fluorescence was correlated to higher complete resection rate, further research is needed to identify patients (or tumors) with more surgery benefits when using the 5-ALA.

## Supporting Information

S1 STROBE Checklist STROBE Statement- checklist. (PDF)
